# The Role of White Matter Disease in Impaired Insight Among Individuals with Cognitive Impairment: Exploring Subtypes and Cognitive Pathways

**DOI:** 10.1002/alz70857_097778

**Published:** 2025-12-24

**Authors:** Chin Yee Cheong, Li Cheng Heng, Chong Jin Ng, Natalie Jian Ting Wee, Pulickal Geoiphy George, Singh Dinesh Rambachan, Philip Lin Kiat Yap

**Affiliations:** ^1^ Khoo Teck Puat Hospital, Singapore, United Kingdom; ^2^ Imperial College London, London, London, United Kingdom; ^3^ Khoo Teck Puat Hospital, Singapore, Singapore; ^4^ Khoo Teck Puat Hospital, Singapore, Singapore, Singapore

## Abstract

**Background:**

Impaired insight poses significant challenges for individuals with cognitive impairment, complicating diagnosis, treatment adherence, and overall care planning. While white matter disease (WMD) is commonly implicated in cognitive decline, its role in influencing insight and the cognitive pathways mediating this relationship remains unclear. This study examines whether WMD affects insight in individuals with cognitive impairment and explores the mediating role of cognitive domains.

**Method:**

133 participants (mean age 75.8 ± 7.6 years) with mild cognitive impairment or dementia were recruited from a memory clinic. White matter hyperintensities (WMH) were visually rated using the Fazekas scale, and insight was assessed using the Clinical Insight Rating Scale. Structural equation modelling (SEM) was employed to evaluate direct and indirect pathways between WMH subtypes, cognitive domains, and insight, while adjusting for covariates.

**Result:**

More than half of the participants (*n* =  89, 66.9%) had partial to complete insight. Multivariate analysis revealed only deep white matter hyperintensities (DWMH) but not periventricular WMH were significantly associated with poor insight (β = 0.29, 95% CI 0.01–0.57). Impaired judgment was strongly associated with poor insight (β = 1.17, 95% CI 0.41–1.94). In the SEM analysis, unadjusted model showed that DWMH was significantly associated with both judgment (β = 0.28, *p* < 0.001) and insight indirectly via judgment (β = 0.15, *p* = 0.031). In the adjusted model, the relationship between DWMH and judgment remained significant (β = 0.15, *p* =  0.018), but the direct effect of DWMH on insight became non‐significant (β = 0.10, *p* =  0.126). FAST staging emerged as a robust predictor of insight (β = 0.34, *p* < 0.001).

**Conclusion:**

The findings suggest DWMH impairs insight indirectly through its impact on judgment, a critical cognitive domain. While dementia severity (FAST) strongly predicts insight, the persistence of the DWMH–judgment relationship implicates structural brain changes driving cognitive deficits that impair insight. As the prefrontal lobes are known to subserve insight, we hypothesize that the disruption of the frontal‐subcortical circuits by DWM lesions could explain the results. Our study highlights the need for clinicians to anticipate potential challenges in judgment and insight among individuals with significant DWMH.

